# Human subject protection and ethical review of research protocol

**DOI:** 10.1186/1742-4690-9-S2-P233

**Published:** 2012-09-13

**Authors:** NM Otuonye, FO Nwaokorie, D Suprumont, T Halidoub, EI Otuonye

**Affiliations:** 1 Nigerian Institute of Medical Research, Yaba, Nigeria; 2 Institute de droit de la Santé, Faculty de droit Neuchâtel, Neuchatel, Switzerland; 3 IRSS-DRO/Centre Muraz 01, Bobo Dioulass, Burkina Faso; 4 Evergreen Chambers Off Ojuelebga, Surulere Lagos, Nigeria

## Background

The purpose of this project was to train health personnel, researchers and IRB members to design protocols with ethical integrity and to approve the conduct of protocols with scientific merit. The burden of TB, HIV and Malaria is heavier in sub - Saharan Africa; hence there are calls for competent review of ethical processes to ensure that research proposals and protocols comply with international standards.

## Methods

Sixty nine medical personnel including Doctors, Nurses, Biomedical Scientists, and Pharmacists, legal officer, clergy, community representative and Researchers were trained on Human subject protection. Participants were trained on Basic principles and international guidelines on bioethics, Basics of research and clinical trials, Ethical review of research protocols, Risk, benefit and inducement. Other slides that were presented included: Standard of care and prevention, research protocol, standard of care, Good participatory practices, Informed consent, HIV treatment, prevention research and monitoring of research. Pre and Post test was used to evaluation participants’ performance.

## Results

The 4 day workshop conducted at Mainland Hospital Yaba, Lagos, Nigeria, from 28th June to 1st July, 2011, adopted a participatory approach including brainstorming, group discussion, slide presentations and case studies. The pre and post-test evaluation indicated that participants’ knowledge improved significantly in conduct of ethically sound research (45.3% vs 65.0%; P=0.001).

## Conclusion

From our experience training and retraining of health personnel and researchers would increase their knowledge and skill to conduct research that is ethically regulated and of international standard. IRB members would improve their ability to understand the operations of research ethics and develop adequate confidence in reviewing research protocols and monitor research activities.

